# Diagnostic accuracy of pooling urine, anorectal, and oropharyngeal specimens for the detection of *Chlamydia trachomatis* and *Neisseria gonorrhoeae*: a systematic review and meta-analysis

**DOI:** 10.1186/s12916-021-02160-9

**Published:** 2021-11-25

**Authors:** Lily Aboud, Yangqi Xu, Eric P. F. Chow, Teodora Wi, Rachel Baggaley, Maeve B. Mello, Christopher K. Fairley, Jason J. Ong

**Affiliations:** 1 College of Medicine and Dentistry, James Cook University, Townsville, Australia; 2 Central Clinical School, Monash University, Melbourne, Australia; 3 Melbourne Sexual Health Centre, Alfred Health, Melbourne, Australia; 4 Centre for Epidemiology and Biostatistics, Melbourne School of Population and Global Health, The University of Melbourne, Melbourne, Australia; 5 Global HIV, Hepatitis and STI Programmes, World Health Organization, Geneva, Switzerland; 6 Faculty of Infectious and Tropical Diseases, London School of Hygiene and Tropical Medicine, London, UK

**Keywords:** Sexually transmitted infections, Chlamydia, Gonorrhoea, Testing, Screening

## Abstract

**Background:**

Screening for *Chlamydia trachomatis* (CT) and *Neisseria gonorrhoeae* (NG) at genital and extragenital sites is needed for most key populations, but molecular diagnostic tests for CT/NG are costly. We aimed to determine the accuracy of pooled samples from multiple anatomic sites from one individual to detect CT/NG using the testing of a single sample from one anatomic site as the reference.

**Methods:**

In this systematic review and meta-analysis, we searched five databases for articles published from January 1, 2000, to February 4, 2021. Studies were included if they contained original data describing the diagnostic accuracy of pooled testing compared with single samples, resource use, benefits and harms of pooling, acceptability, and impact on health equity. We present the pooled sensitivities and specificities for CT and NG using a bivariate mixed-effects logistic regression model. The study protocol is registered in *PROSPERO*, an international database of prospectively registered systematic reviews (CRD42021240793). We used GRADE to evaluate the quality of evidence.

**Results:**

Our search yielded 7814 studies, with 17 eligible studies included in our review. Most studies were conducted in high-income countries (82.6%, 14/17) and focused on men who have sex with men (70.6%, 12/17). Fourteen studies provided 15 estimates for the meta-analysis for CT with data from 5891 individuals. The pooled sensitivity for multisite pooling for CT was 93.1% [95% confidence intervals (CI) 90.5–95.0], *I*^*2*^=43.3, and pooled specificity was 99.4% [99.0–99.6], *I*^*2*^=52.9. Thirteen studies provided 14 estimates for the meta-analysis for NG with data from 6565 individuals. The pooled sensitivity for multisite pooling for NG was 94.1% [95% CI 90.9–96.3], *I*^*2*^=68.4, and pooled specificity was 99.6% [99.1–99.8], *I*^*2*^=83.6. Studies report significant cost savings (by two thirds to a third).

**Conclusion:**

Multisite pooled testing is a promising approach to improve testing coverage for CT/NG in resource-constrained settings with a small compromise in sensitivity but with a potential for significant cost savings.

**Supplementary Information:**

The online version contains supplementary material available at 10.1186/s12916-021-02160-9.

## Background

Data from the 2021 WHO global progress report on HIV, viral hepatitis, and sexually transmitted infections indicate a global incidence of 128 million new chlamydia and 82 million new gonorrhoea cases in 2020 [1]. Representing neglected pandemics, these infections cause a significant global disease burden. There are population groups who are disproportionately affected by STIs, including men who have sex with men (MSM), sex workers (SW) and their clients, transgender people (TG), adolescent girls and young women (AGWY), and pregnant women [1]. There is also a high prevalence and incidence of STIs among people taking pre-exposure prophylaxis for HIV (PrEP) [2] and young women attending contraceptive services in East and Southern Africa [3], many of which would have been missed if syndromic STI management had been used. This has led to a push for greater access to aetiological testing in PrEP programmes [4]. Major gaps persist in the availability of diagnosis and treatment for CT/NG, with STI programmes and services generally underfunded despite high levels of morbidity and mortality.

To control STIs, earlier detection and treatment are needed. Yet, a significant challenge is that most STIs are asymptomatic and require testing to identify infection. Undiagnosed and untreated STIs can lead to onward transmission and morbidity such as reproductive organs inflammation, reproductive morbidity and infertility, and vertical transmission to neonates. Viral and bacterial STIs can increase the risk of acquiring HIV, as increased viral loads of HIV can be found in genital tracts during STI coinfection [5]. Furthermore, inappropriate management of gonococcal infection may accelerate the emergence of multidrug-resistant NG [6]. This underscores the need for aetiological diagnosis to optimise STI management.

An aetiological diagnosis that tests all appropriate anatomic sites is needed. Evaluation for CT/NG at extragenital sites is critical for some population groups (e.g., MSM, SW, TG), as a significant proportion of infections would be missed if only genital testing were undertaken [7]. Studies have demonstrated that up to two thirds of NG cases would be missed if only urethral or urine samples were tested in MSM [8]. CT and NG are highly transmissible and often asymptomatic, and early detection relies on regular and comprehensive testing of multiple anatomic sites for those at higher risk [8, 9]. Since 2010, the US Centres for Disease Control (CDC) have recommended using NAAT to test for extragenital CT and NG, as molecular testing improved sensitivity compared to culture [10]. The current Australian STI management guidelines recommend that pharyngeal, anorectal, and urethral testing is undertaken in asymptomatic MSM [9]. In women, a pharyngeal swab and anorectal swab are also recommended depending on reported sexual practices [9].

Whilst testing from three anatomic sites individually would be ideal, the increased cost of NAAT over culture is a major limitation [11], especially in low- and middle-income countries and other resource-limited settings. Furthermore, testing multiple anatomic sites separately can increase costs and workload, especially when implementing testing at or near the point of care. Several studies have investigated the pooling of specimens from triple anatomic sites from a single individual, but its accuracy varied across studies [12–14]. Currently, there is no clear consensus whether pooling has adequate accuracy for populations at higher risk. Furthermore, a 2018 UK study reported that most clinicians regarded the existing evidence of pooling as insufficient to justify implementation in clinical practice [15]. Since 2018, many more studies have been published, and a critical appraisal of all available evidence is helpful to guide future guidelines and practices.

If pooling of samples from the pharynx, urethra/endocervix, and anorectum within a single individual is demonstrated to be both highly sensitive and specific for *Chlamydia trachomatis* and *Neisseria gonorrhoea* detection, this could provide significant cost savings and influence national guidelines and clinical practice. The primary aim of this systematic review was to review and critically appraise the existing evidence regarding the diagnostic accuracy of pooled samples from triple anatomic sites of one individual for the testing of CT/NG using a single sample from one anatomic site as the reference. The secondary aims were to assess the cost impact of using pooled specimens, the patient and provider acceptability of the pooled sample approach, and the effects of pooled testing on health equity.

## Methods

### Search strategy and selection criteria

#### Inclusion and exclusion criteria

To be included in this systematic review and meta-analysis, the study contained primary data assessing at least one of the following outcomes: the diagnostic accuracy of the pooled testing approach (index test) compared with a single sample (reference standard), resource use, benefits and harms, patient or provider acceptability, and impacts on health equity. A study was excluded if it was a duplicate, full text not available, or irrelevant to the outcomes of interest.

### Search strategy

Five databases (MEDLINE, Embase, CINAHL, CABI Global Health, Web of Science) were searched for articles published anytime from January 1, 2000, to the search date of February 4, 2021, limited to the English language. The search strategies looked for information on pooling samples for STI testing from three anatomic sites (urethra/endocervix, anorectal, and pharynx). The search strategy was refined with the research team and librarian using different combinations of key terms until the results retrieved reflected the scope of the project. Further details of the search strategy are provided in Additional file 1: Appendix 1.

### Study selection

**Fig. 1:**
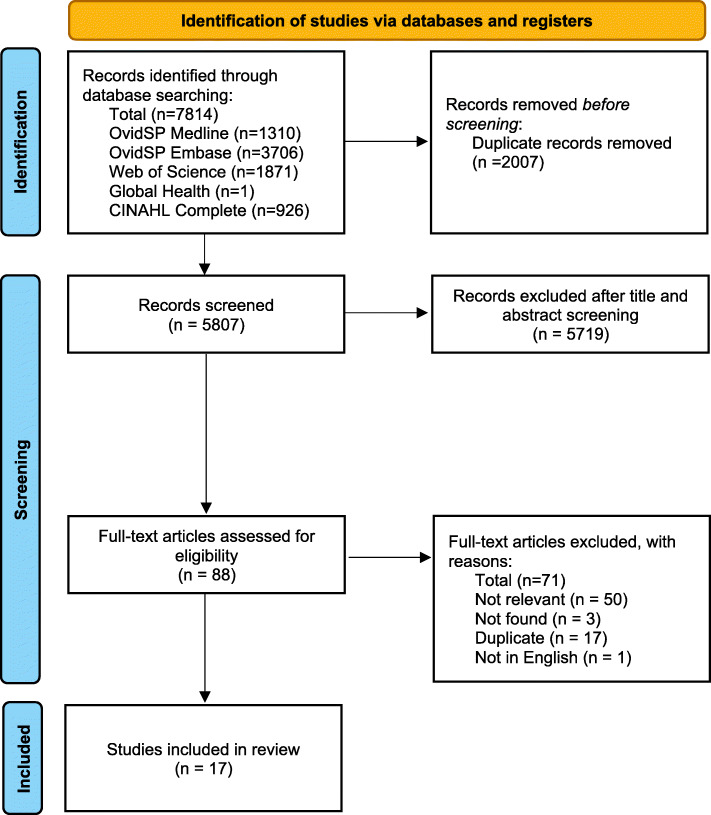
PRISMA flowchart

Two researchers (LA, YX) independently reviewed the titles and abstracts using Covidence, with the resolution of conflicts by a third researcher (JO). The selection process is summarised in the PRISMA study flow diagram (Fig. 1).

### Data analysis

#### Data extraction

Two researchers (LA, YX) independently extracted data with a third reviewer (JO) resolving all conflicts. An electronic data extraction form was used to extract information from each study, and this included the author, publication year, country, study year, study type, study population, sample size, study settings, study aims, method of pooling, and pooling results (true positive, false positive, true negative, false negative) compared to the reference standard, resource use, acceptability, impacts on health equity, benefits and harms, and follow-up actions with results of pooled testing. We contacted several authors to clarify the performance accuracy reported in their studies [13, 16].

#### Risk of bias assessment

Included studies that contained information about the performance of multisite pooled testing were evaluated using the QUADAS-2 checklist by two researchers (RX and LA). We assessed the certainty of the evidence using the GRADE [17, 18].

#### Data analysis

Descriptive statistics were used to summarise the characteristics of included studies. We used a bivariate mixed-effects logistic regression model in STATA version 17 (StataCorp. 2019. *Stata Statistical Software: Release 17*. College Station, TX: StataCorp LLC). Statistical heterogeneity between studies was assessed with the *I*^*2*^ statistic. Random-effects meta-regression models were conducted to explore study-level factors to explain the heterogeneity observed. Deek’s test was used to evaluate for small-study effects.

We reported the pooled sensitivity, specificity, positive and negative likelihood ratios, and diagnostic odds ratio. The positive likelihood ratio expresses how many times more likely people with the condition receive a positive test result than those who do not have the condition. In contrast, the negative likelihood ratio expresses how likely it is that people with the condition will receive a negative test result than those who do not have the condition. The inverse of the negative likelihood ratio (1/LR-) can be compared with the positive likelihood ratio to indicate whether the positive or negative test result has a greater impact on the odds of disease. We also present the summary receiver operating characteristic (SROC) curve from the hierarchical summary receiver operating characteristic (HROC) model, the prediction region (i.e. for the forecast of the true sensitivity and specificity in a future study). Plotting the summary operating point and its confidence region allowed us to graphically display the trade-off between sensitivity and specificity. Forest plots were used to show within-study estimates and confidence intervals for sensitivity and specificity separately. We report our findings using the PRISMA checklist.

#### Registration

The systematic review was conducted with the guidance of the Cochrane Handbook 5.1. The study protocol is registered in *PROSPERO*, an international database of prospectively registered systematic reviews (CRD42021240793).

#### Role of the funding source

WHO technical staff were involved in the study design, result interpretation, and decision to submit the study for publication.

## Results

We identified a total of 7814 records using our search strategies, 88 full texts were examined, and 17 studies were eligible and included in the analysis (Fig. 1).

### Study characteristics (Table 1)

**Fig. 2:**
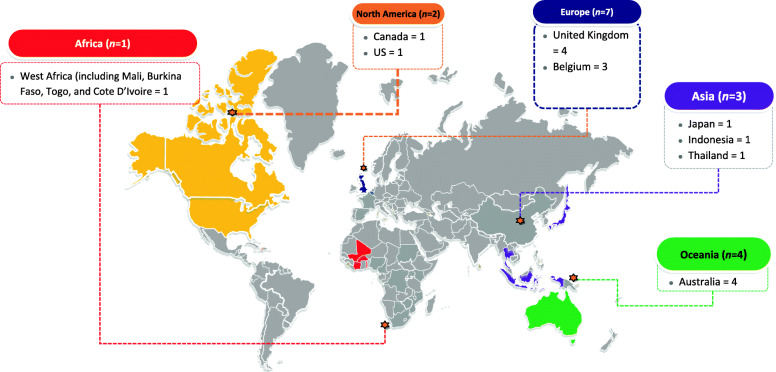
Countries of studies with an evaluation of multisite pooled testing for CT and NG (*N*=17)

Most studies were conducted in high-income countries (82.6%, 14/17) (Fig. 2) and in community outpatients (47.1%, 8/17). MSM were the most frequently studied population (70.6%, 12/17). The majority of studies included both clinician and self-collected samples (29.4%, 5/17), followed by self-collected samples (23.5%, 4/17) and health provider collected samples (11.8%, 2/17). Fourteen out of 17 studies reported the diagnostic accuracy of multisite pooled testing and were included in the meta-analysis.

**Table 1:** Characteristics of 17 included studies

Study characteristics	Total (***N***=17)
Country income level* ^	***n*** **(%)**
*High*	14 (82.4)
*Middle*	2 (11.8)
*Low*	1 (5.9)
Settings*	
*Primary care*	4 (23.5)
*Youth health centres*	1 (5.9)
*Hospital*	1 (5.9)
*Community outpatient clinic*	8 (47.1)
*STI clinic*	4 (23.5)
*Sex on premises venue*	1 (5.9)
*Not specified*	2 (11.8)
Populations*	
*MSM*	12 (70.6)
*Cis-women*	3 (17.6)
*Transgender women*	2 (11.8)
*Female sex workers*	1 (5.9)
*Not specified*	2 (11.8)
**Pharyngeal and anorectal testing collected by**	
*Patient only*	4 (23.5)
*Provider only*	2 (11.8)
*Both*	5 (29.4)
*Not specified*	6 (35.3)
**Outcomes addressed**	
*Diagnostic accuracy of triple site pooling*	14 (82.4)
*Resource use*	4 (23.5)
*Acceptability*	4 (23.5)
*Harms and benefits*	6 (35.3)
*Health equity*	9 (52.9)

*Some studies contained more than one population/setting/country

^As per the New World Bank current 2021 fiscal year [19]

### Diagnostic accuracy of multisite pooled testing for chlamydia

**Fig. 3:**
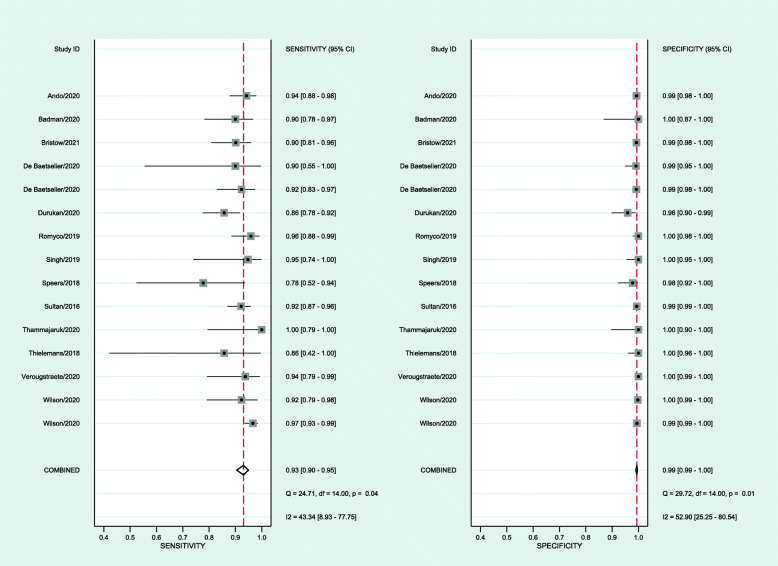
Forest plot of the sensitivity and specificity of multisite pooled testing for chlamydia

Fourteen studies provided 15 estimates for the meta-analysis with data from 5891 individuals. Table 2 and Fig. 3 show that the pooled sensitivity was 93.1%, and pooled specificity was 99.4%. Additional file 1: Supplementary Figure 1 shows the receiver operating curve, demonstrating the high accuracy of multisite pooled testing. Publication bias was unlikely (*p*=0.07, Additional file 1: Supplementary Figure 2). Additional file 1: Supplementary Table 1 summarises the meta-regression results showing significant heterogeneity related to MSM populations with slightly lower sensitivity compared to non-MSM populations (−0.8% (95% CI −1.6 to 0)). Additional file 1: Supplementary Table 2 demonstrates the impact on positive and negative predictive values when the background prevalence of chlamydia changes. The certainty of the evidence is moderate because most studies had patient selection bias and some had the potential for flow and timing bias (Additional file 1: Supplementary Tables 3 and 4, Additional file 1: Supplementary Figure 3).

**Table 2:** Diagnostic accuracy of multisite pooled testing for chlamydia and gonorrhoea

	Chlamydia (***N***=5891)	Gonorrhoea (***N***=6565)
Pooled sensitivity	93.1% [95% CI 90.5−95.0, *I*^*2*^=43.3, *p*<0.001]	94.1% [95% CI 90.9−96.3, *I*^*2*^=68.4, *p*<0.001]
Pooled specificity	99.4% [95% CI 99.0−99.6, *I*^*2*^=52.9, *p*<0.001]	99.6% [95% CI 99.1−99.8, *I*^*2*^=83.6, *p*<0.001]
Diagnostic odds ratio	2181 [1013−4696]	4190 [1435−12237]
Positive likelihood ratio	152 [88−263]	246 [102−593]
Negative likelihood ratio	0.07 [0.05−0.10]	0.06 [0.04−0.09]
Inverse negative likelihood ratio	14 [10−20]	17 [11−27]

*95% CI* 95% confidence intervals

#### Diagnostic accuracy of multisite pooled testing for gonorrhoea

**Fig. 4:**
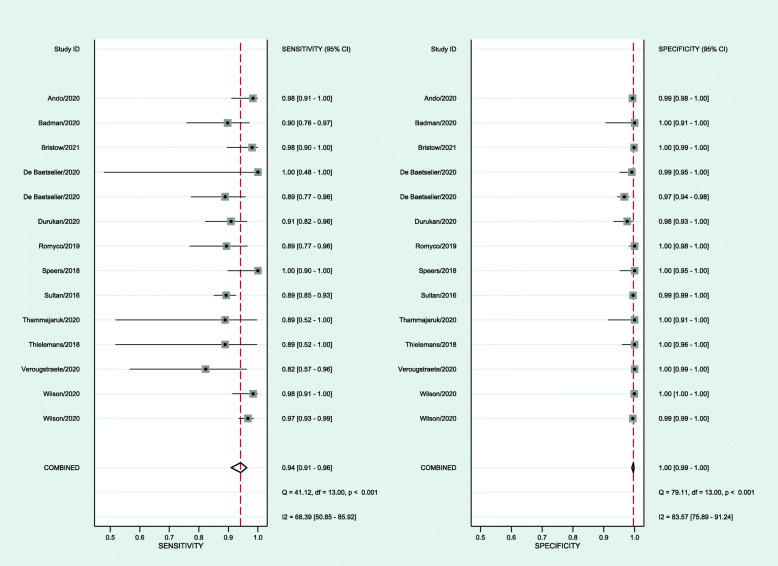
Forest plot of the sensitivity and specificity of multisite pooled testing for gonorrhoea

Thirteen studies provided 14 estimates for the meta-analysis with data from 6565 individuals. The pooled sensitivity was 94.1%, and pooled specificity was 99.6%. Additional file 1: Supplementary Figure 4 shows the receiver operating curve, demonstrating the high accuracy of multisite pooled testing. Publication bias was unlikely (*p*=0.18, Additional file 1: Supplementary Figure 5). Additional file 1: Supplementary Table 5 summarises the meta-regression results with no significant differences in the study population, study population size, country-income level, sample collection, or publication year. Figure 4 is the Forest plot of the sensitivity and specificity of multisite pooled testing for gonorrhoea. Additional file 1: Supplementary Table 6 demonstrates the impact on positive and negative predictive values when the background prevalence of gonorrhoea changes. The certainty of the evidence is low for sensitivity because most studies had patient selection bias and lower sensitivity was noted for detecting pharyngeal gonorrhoea (Additional file 1: Supplementary Tables 3 and 7). The certainty of the evidence is moderate for specificity because most studies had patient selection bias and some had the potential for flow and timing bias.

We provide further details about the methodology of multisite pooled testing (Additional file 1: Supplementary Table 8). There, we also provide further details on the actions taken post-positive pooled test result and the subanalysis on the diagnostic accuracy of pharyngeal pooled testing.

#### Resource use

Four studies commented on the cost saving aspects of pooled testing from triple anatomic sites. Verougstraete et al. [20] conducted a prospective study between February 2018 and July 2019 involving 501 female sex workers in Belgium. Their study demonstrated a 35% decrease in reagent costs and lab technician time when using pooled testing. This was calculated using the obtained prevalence of 6.5% and 3.5% for CT and NG, respectively. Sultan et al. [13] conducted a study of 1064 MSM attending UK sexual health clinics and hospital sites between October 2012 and August 2013. Whilst they acknowledged that the costs of each assay varied according to different laboratories, they proposed that pooled testing offers cost savings of up to two thirds of the costs of the assays alone, as well as savings in consumables, processing time, and clinical pathway efficacy. De Baetselier et al. [21] assessed the efficacy of pooled testing among 497 MSM in four West African countries. They demonstrated a 56% decrease in cost when un-pooling of triple-site pooling was only undertaken when the pooled sample result was invalid. If both invalid and CT/NG positive pooled samples were un-pooled, there was a total decrease in cost by 30%. We identified one cost-effectiveness study by Wilson et al. [22] of MSM attending sexual health clinics in the UK (2015–2016). They reported that using a willingness to pay threshold of £60 per person tested, pooled testing had a 100% probability of being cost-effective. Compared with individually analysed samples, pooled testing saved £13.37 to £18.22 per individual tested depending on the symptom status or population group (MSM, women from the general population).

#### Potential harms

Six studies conducted in the UK, Australia, West Africa, and Belgium discussed the possible adverse events of multisite pooled testing. One universal issue was that pooled testing failed to provide site-specific information without retesting individuals, potentially limiting site-specific treatment choices [13, 15, 20, 21, 23, 24]. For example, if the details of site-specific infection were available, anorectal chlamydia would be treated with doxycycline instead of azithromycin [13, 20]. Speers et al. also discussed that the sensitivity of retesting the same individual samples could be reduced due to the elution of the swabs [23]. If a laboratory method was able to test for the adequacy of the sample, pooling samples would not allow for testing the adequacy of individual samples.

#### Provider and patient acceptability

Shaw’s et al. reported that 84% (41/49) of sexual health clinicians in England considered the most significant benefit of pooling was cost savings [15]. The greatest barriers were lack of supportive evidence, lack of national guidance, loss of infection site information, and a perceived reduction in sensitivity or specificity [15]. In addition, most (77%, 40/52) clinicians requested more validation studies on the diagnostic accuracy, 75% (39/52) wanted clinical guidelines of pooling, and 48% (25/52) of clinicians required further cost analysis.

Two of four studies provided data about participants’ acceptability of self-collected specimen as part of multisite pooled testing [13, 25]. Both studies reported self-sampling was acceptable. Sultan et al. showed most participants found it easy to collect both anorectal and pharyngeal samples (93% and 89%, respectively), and the majority (91%) were confident to take self-collected samples [13]. Similarly, almost all participants (≥97%) in Chernesky’s study found steps to self-collect vaginal swabs easy to follow after receiving visual and verbal instructions in the clinic [25]. In addition to self-collecting, participants in Chernesky’s study also self-pooled the samples. However, only two samples (urine and vaginal) were taken, inconsistent with the triple site pooling method this systematic review focused on. Collecting or pooling double site specimens could be easier and different from triple site collecting or pooling. No quantitative data on the acceptability of the steps of self-pooling was provided in Chernesky’s study.

#### Impact on health equity

Eight studies discussed the health equity impacts pooling could bring by increasing the testing coverage [15, 20, 21, 23, 26–28], especially for asymptomatic individuals who would not have been tested otherwise in low- and middle-income countries such as countries in West Africa [21]. De Baetselier et al. [21] suggested that pooled testing should be incorporated in PrEP programs in resource-limited countries due to the potential to decrease HIV transmission. In addition, cost savings via pooled testing would allow more people to be tested or for those at higher risk of infection to be tested more frequently within a limited budget.

## Discussion

Our systematic review appraises the current evidence of the diagnostic accuracy of pooling from triple anatomic sites for CT/NG testing. The combined sensitivity for CT and NG were 93.1% and 94.1%, respectively. The combined specificity for CT and NG was 99.4% and 99.6%, respectively. Programmes and services will need to assess whether the small decrease in sensitivity associated with multisite pooled testing warrants the substantial cost savings and potential improvements in health inequity.

The benefit of pooling will be restricted to testing for those reporting extragenital sexual practices. In a UK study involving MSM and women from the general population, Wilson et al. showed that pooling could save up to £18.22 per individual tested, a significant cost saving when multiplied by the number of people tested at the population level [22]. Viewed a different way, if there is a fixed budget, pooling could increase the numbers of people tested and the frequency of testing for those at higher risk [21, 22]. In particular, this could enable greater access to testing, including more regular testing for those with a higher risk of STIs. This includes individuals taking PrEP, where most national guidelines recommend routine triple-site testing for users [29]. Pooled testing encourages multisite STI testing at a lower expense, which is more effective in detecting CT/NG infections than the single-site testing in which many extragenital infections would be missed [20, 27]. The prevalence at the extragenital site is usually higher than the genital site, and they are usually asymptomatic; therefore, both anorectal and pharyngeal specimens are recommended to be included in testing [30]. Verougstraete et al. [20] reported that 40% of CT and 60% of NG infections would have been missed if only genital samples were tested. Similarly, if only urine samples were tested among 76 participants enrolled in Badman et al. [27], 82% of CT and 85% of NG infections would be missed. Furthermore, the background prevalence of CT/NG influences the cost-effectiveness of pooled testing. Pooled testing would demonstrate higher cost savings in low prevalence settings/populations or high prevalence settings/populations if retesting was not required when a uniform treatment protocol is used as recommended by WHO as the first line for both CT/NG [31]. In addition to the potential for cost savings, participants reported high acceptability to self-collecting samples, and we found no significant difference in the diagnostic accuracy compared to clinician collected samples. Therefore, self-collected pooled specimens could further reduce barriers to STI testing [20]. By ensuring multisite testing among relevant populations, pooling can expand the testing coverage and increase the frequency of testing among those at higher risk of infection. This could potentially limit the CT/NG pandemic and reduce HIV transmission.

There are several potential limitations to multisite pooled testing. The lack of anatomic-site specific results was a common concern in the studies reviewed and suggests the need to retest those with positive results. Although some guidelines have some differences in the treatment recommended for CT and NG depending on the site of infection, in the current WHO guidelines, the first therapeutical line recommended can be the same for infections regardless of anatomic site [31, 32]. Using pooled testing may impair epidemiological data collection for reporting CT/NG infections, as the site of infection would not be known without testing individual sites. Whilst demonstrated to reduce the total costs of testing, pooled testing may still not be affordable in resource-constrained settings without established STI testing infrastructure or not adopting molecular point-of-care testing for STIs or other infections such as tuberculosis or to measure HIV viral load. Not only does pooled testing require access to NAAT, but it also requires laboratory processes for combining samples before testing. Considerations include the transport and handling of samples, mixing of samples, the amount of diluent used, potential contamination of samples, laboratory staff training, and the storage of individual samples for re-testing if required [15, 20]. Finally, with lower bacterial load in the oropharynx compared to the genital and anorectal sites [33], there is a potential for pooled testing to miss oropharyngeal infections [13, 26, 27, 34]. Future studies should investigate how to further optimize detection of oropharyngeal infections.

Limitations of this review include the high variation in the pooling method and the assays used for each study. We are therefore unable to comment on an optimal method of pooling. This review is also limited by our exclusion of studies in languages other than English. Additionally, our data was mainly gathered from high-income countries (82.6%, 14/17) and used MSM as a study population (70.6%, 12/17), limiting the generalisability of this review to other populations and low- and middle-income countries. Studies were conducted in the community outpatient setting (47.1%, 8/17), where symptomatic people are more likely to attend and therefore increase the likelihood of returning a positive result.

Our systematic review has highlighted areas for further research, including the feasibility and patient acceptability of self-collected sampling combined with self-pooling and feasibility from a laboratory staff perspective. Appropriately powered studies are required for the evaluation of pharyngeal NG and CT sensitivities. Further studies should also be undertaken to assess the acceptability of pooling by providers considering the synthesis of evidence and approvals from peak bodies such as the WHO. Future studies should be conducted to test the impact of urine volume and order of swabbing on the diagnostic accuracy of pooled samples. In addition, there is a need for more implementation studies to assess any treatment delays or additional costs associated with retesting individual samples if anatomic site-specific information is needed to guide treatment.

## Conclusions

Our systematic review and meta-analysis found that multisite pooled testing for CT/NG is a sensitive and specific method. Multisite pooled testing for CT/NG can improve access to more individuals for testing and for relevant populations to be regularly tested for extragenital site infection.

## Supplementary Information

**Additional file 1: Appendix 1.** Search strategy. **Appendix 2.** Further details of multisite pooled sampling for chlamydia and gonorrhoea. **Supplementary Figure 1.** Receiver operating characteristic (ROC) curve for multisite pooled testing for chlamydia. **Supplementary Figure 2.** Assessment for small study effects for multisite pooled testing for chlamydia. **Supplementary Figure 3.** Risk of bias summary as percentage. **Supplementary Figure 4.** Receiver operating characteristic (ROC) curve for multisite pooled testing for gonorrhoea. **Supplementary Figure 5.** Assessment for small study effects for multisite pooled testing for gonorrhoea. **Supplementary Table 1.** Meta-regression of the accuracy of multisite pooled testing for chlamydia according to study characteristics. **Supplementary Table 2.** The positive predictive value (PPV) and negative predictive value (NPV) for multisite pooled testing for chlamydia, over a range of background prevalence of chlamydia. **Supplementary Table 3.** Risk of Bias summary. **Supplementary Table 4.** GRADE table for multisite pooled testing for chlamydia. **Supplementary Table 5.** Meta-regression of the accuracy of multisite pooled testing for gonorrhoea according to study characteristics. **Supplementary Table 6.** The positive predictive value (PPV) and negative predictive value (NPV) for multisite pooled testing for gonorrhoea, over a range of background prevalence of gonorrhoea. **Supplementary Table 7.** GRADE table for multisite pooled testing for gonorrhoea. **Supplementary Table 8.** Study characteristics, methods of pooling, reported sensitivity and specificity of multisite pooled testing. References.

## Data Availability

All data generated or analysed during this study are included in this published article and its supplementary information files.
