# Supplementary material for: Diagnostic accuracy of pooling urine, anorectal, and oropharyngeal specimens for the detection of Chlamydia trachomatis and Neisseria gonorrhoeae: a systematic review and meta-analysis
Source: BMC Med. 2021 Nov 25;19:285. doi: 10.1186/s12916-021-02160-9 (PMC8614052; doi:10.1186/s12916-021-02160-9)
Supplement: Supplementary file 1 — Additional file 1: Appendix 1. Search strategy. Appendix 2. Further details of multisite pooled sampling for chlamydia and gonorrhoea. Supplementary Figure 1. Receiver operating characteristic (ROC) curve for multisite pooled testing for chlamydia. Supplementary Figure 2. Assessment for small study effects for multisite pooled testing for chlamydia. Supplementary Figure 3. Risk of bias summary as percentage. Supplementary Figure 4. Receiver operating characteristic (ROC) curve for multisite pooled testing for gonorrhoea. Supplementary Figure 5. Assessment for small study effects for multisite pooled testing for gonorrhoea. Supplementary Table 1. Meta-regression of the accuracy of multisite pooled testing for chlamydia according to study characteristics. Supplementary Table 2. The positive predictive value (PPV) and negative predictive value (NPV) for multisite pooled testing for chlamydia, over a range of background prevalence of chlamydia. Supplementary Table 3. Risk of Bias summary. Supplementary Table 4. GRADE table for multisite pooled testing for chlamydia. Supplementary Table 5. Meta-regression of the accuracy of multisite pooled testing for gonorrhoea according to study characteristics. Supplementary Table 6. The positive predictive value (PPV) and negative predictive value (NPV) for multisite pooled testing for gonorrhoea, over a range of background prevalence of gonorrhoea. Supplementary Table 7. GRADE table for multisite pooled testing for gonorrhoea. Supplementary Table 8. Study characteristics, methods of pooling, reported sensitivity and specificity of multisite pooled testing. References. [file 12916_2021_2160_MOESM1_ESM.zip › Additional File 1 CLEANR2.pdf]

TABLE OF CONTENTS

Appendix 1 Search Strategy 2

Appendix 2: Further details of multisite pooled sampling for chlamydia and gonorrhoea ...6

Appendix 3: Supplementary Figures and Tables 8

Supplementary Figure 1. Receiver operating characteristic (ROC) curve for multisite pooled testing for chlamydia. 8

Supplementary Figure 2. Assessment for small study effects for multisite pooled testing for chlamydia. 9

Supplementary Figure 3. Risk of Bias Summary as percentage 10

Supplementary Figure 4. Receiver operating characteristic (ROC) curve for multisite pooled testing for gonorrhoea. 11

Supplementary Figure 5. Assessment for small study effects for multisite pooled testing for gonorrhoea. 12

Supplementary Table 1. Meta-regression of the accuracy of multisite pooled testing for chlamydia according to study characteristics 13

Supplementary Table 2. The positive predictive value (PPV) and negative predictive value (NPV) for multisite pooled testing for chlamydia, over a range of background prevalence of chlamydia. 14

Supplementary Table 3. Risk of Bias Summary 15

Supplementary Table 4. GRADE table for multisite pooled testing for chlamydia 17

Supplementary Table 5. Meta-regression of the accuracy of multisite pooled testing for gonorrhoea according to study characteristics 18

Supplementary Table 6. The positive predictive value (PPV) and negative predictive value (NPV) for multisite pooled testing for gonorrhoea, over a range of background prevalence of gonorrhoea. 19

Supplementary Table 7. GRADE table for multisite pooled testing for gonorrhoea 20

Supplementary Table 8. Study characteristics, methods of pooling, reported sensitivity and specificity of multisite pooled testing 21

Appendix 1 Search Strategy

1. Search methodology

Five databases were searched on 04 February 2021. The search strategies looked for information on the pooling of STI samples for STI screening from three anatomic sites (urethra, anorectum and pharynx). The search limits were from 2000 to current and English language. The search strategy was refined with the research team until the results retrieved reflected the scope of the project. The final Medline search was amended to run across the other databases except for CINAHL Complete, in which we utilised a simplified search strategy to attain an appropriate size of references.

The database searched were:

1. OvidSP Medline and In-Process & Other Non-Indexed Citations and Daily, 1946 to February 1, 2021.
2. OvidSP Embase, 1974 to February 1, 2021.
3. EBSCO CINAHL Complete, complete database
4. CABI Global Health, 1910 to February 1, 2021
5. Web of science core collection:
   a) Science Citation Index Expanded (1900-present)
   b) Social Sciences Citation Index (1900-present)
   c) Arts & Humanities Citation Index (1975-present)
   d) Conference Proceedings Citation Index- Science (1990-present)
   e) Conference Proceedings Citation Index- Social Science & Humanities (1990-present)
   f) Book Citation Index– Science (2005-present)
   g) Book Citation Index– Social Sciences & Humanities (2005-present)
   h) Emerging Sources Citation Index (2015-present)
   i) Current Chemical Reactions (1985-present)
   j) Index Chemicus (1993-present)

2. Search results

Database name	Endnote importer order	Number of references before deduplication	Number of references after deduplication (removed)
OvidSP Medline	1	1310	1310
OvidSP Embase	2	3706	2704
Web of Science	3	1871	980
Global Health	4	1	1
CINAHL complete	5	926	812
Total			5807

3. Search strategies

3.1 OvidSP Medline

Database name	Medline
Database platform	OvidSP
Dates of database coverage	2000 to Feb 01 2021
Date searched	04/02/2021
Searched by	RX
Number of hits	1310

1. (pool*or mixed* or '3 in 1').mp. (632346)

2. (chlamydia* or trachomatis).mp. (31972)

3. (neisseria or gonorrh?ea*).mp. (39190)

4. (sti or std or sexually transmitted disease* or sexually transmitted infecti* or sexually transmissible infecti* or sexually transmissible disease*).mp. (53716)

5. (screen* or diagnos* or detect* or Nucleic Acid Amplification or PCR or test or testing).mp. (8960816)

6. (accura* or sensitiv* or specific* or feasib* or acceptab* or effective*).mp (6915087)

7. 2 or 3 or 4 (107493)

8. 5 or 6 (13204457)

9. 1 and 7 and 8 (1734)

10. limit 9 to (english language and yr="2000 -Current") (1310)

3.2 OvidSP Embase

Database name	Embase
Database platform	OvidSP
Dates of database coverage	2000 to Feb 01 2021
Date searched	04/02/2021
Searched by	RX
Number of hits	3706

1. (pool*or mixed* or “3 in 1”).mp. (864882)
2. (chlamydia* or trachomatis).mp. (45849)
3. (neisseria or gonorrh?ea*).mp. (58281)
4. (sti or std or sexually transmitted disease* or sexually transmitted infecti* or sexually transmissible infecti* or sexually transmissible disease*).mp. (81114)
5. (screen* or diagnos* or detect* or Nucleic Acid Amplification or PCR or test or testing).mp. (12605409)
6. (accura* or sensitiv* or specific* or feasib* or acceptab* or effective*).mp (9031288)
7. 2 or 3 or 4 (154811)
8. 5 or 6 (17776719)
9. 1 and 7 and 8 (4351)
10. limit 9 to (english language and yr="2000 -Current") (3706)

3.3 Web of Science

Database name	Web of Science
Database platform	Clarivate Analytics Web of Science Core Collection
Dates of database coverage	2000 to Feb 01 2021
Date searched	04/02/2021
Searched by	RX
Number of hits	1871

#1 TOPIC: (pool* or mixed or "3 in 1") (1348108)

#2 TOPIC: (chlamydia* or trachomatis or neisseria or gonorrh?ea* or sti or std or sexually transmitted disease* or sexually transmitted infecti* or sexually transmissible infecti* or sexually transmissible disease*) (75166)

#3 TOPIC: (screen* or diagnos* or detect* or Nucleic Acid Amplification or PCR or test or testing or accura* or sensitiv* or specific* or feasib* or acceptab* or effective*) (16,327,124)

#4 #1 and #2 and #3 (1871)

3.4 CINAHL complete

Database name	CINAHL complete
Database platform	EBSCOhost
Dates of database coverage	2000 to Feb 01 2021
Date searched	04/02/2021
Searched by	RX
Number of hits	926

S1. TX pool* (124171)

S2. TX chlamydia* or trachomatis or neisseria or gonorrh?ea* (15337)

S3. TX screen* or diagnos* or detect* or test or testing or accura* or sensitiv* or specific* or feasib* or acceptab* or effective* (3118460)

S4. S1 AND S2 AND S3 (926)

3.5 Global Health

Database name	Global Health
Database platform	CABI
Dates of database coverage	2000 to Feb 01 2021
Date searched	04/02/2021
Searched by	RX
Number of hits	1

1. pool* or mixed or "3 in 1" (323487)

2. TX chlamydia* or trachomatis or neisseria or gonorrh?ea* or sti or std or sexually transmitted disease* or sexually transmitted infecti* or sexually transmissible infecti* or sexually transmissible disease* (625)

3. screen* or diagnos* or detect* or Nucleic Acid Amplification or PCR or test or testing or accura* or sensitiv* or specific* or feasib* or acceptab* or effective* (128409)

4. 1 AND 2 AND 3 (1)

Appendix 2: Further details of multisite pooled sampling for chlamydia and gonorrhoea

Action post positive pooled test

Only five studies[1-5] retested the same positive individual samples to determine the site-specific infection, yet the follow-up of site-specific treatment for participants was not discussed. In addition to unpooled positive samples, invalid samples were also retested in the study of de Baetselier et al.[1] whilst in Wilson's study, both indeterminate and positive samples were individually retested.[4] PCR was undertaken to identify the gene mutations associated with AMR in positive NG samples in Chernesky's study, but no further information of treatments was available.[6]

Testing for LGV follows the initial detection of chlamydia by standard assays. Pooling should not influence decisions about whether a positive chlamydia samples in an individual is then tested for LGV. This decision will be based on local epidemiology.

Pharyngeal Sampling

Four studies among MSM provided information specifically on the sensitivity of detecting pharyngeal pathogens using pooled testing versus the standard of care (i.e. testing of individual swabs). Sensitivities for pharyngeal CT of 100% were noted by Badman et al.,[7] Dean et al.,[8] and Thammajaruk et al.[9] However, Sultan et al.[10] reported a 69.2% sensitivity for pharyngeal CT among 1064 MSM in the UK. The authors hypothesise that the lower performance of pooled testing in detecting pharyngeal infections may be due to inadequate swabbing technique in self-sampling or the lower organism loads seen in pharyngeal infection. However, they also reported that most NG infections were pharyngeal, with a sensitivity of 89.1% for pharyngeal gonorrhoea. This discordance requires further investigation in studies appropriately powered to detect the performance of pooled testing at different anatomical sites. There were lower sensitivities for pharyngeal NG in all studies: 78%,[8] 80%,[7] 89%[10] and 93%.[9] The differences in sensitivity were hypothesised to be attributed to the lower bacterial load in the pharynx compared to other anatomic sites.[7,9,10]

When pharyngeal infections were excluded, Sultan et al.[10] demonstrated an increase in overall pooled testing sensitivity from 91.9% to 94.2% for CT, and 89.9% to 94.4% for NG. Dean et al.[8] similarly demonstrated an increase in sensitivity when pharyngeal infections were excluded,

with pooled testing sensitivity increasing from 98% to 100% for CT infections, and 93.2% to 100% for NG infections.

Appendix 3: Supplementary Figures and Tables

Supplementary Figure 1. Receiver operating characteristic (ROC) curve for multisite pooled testing for chlamydia.

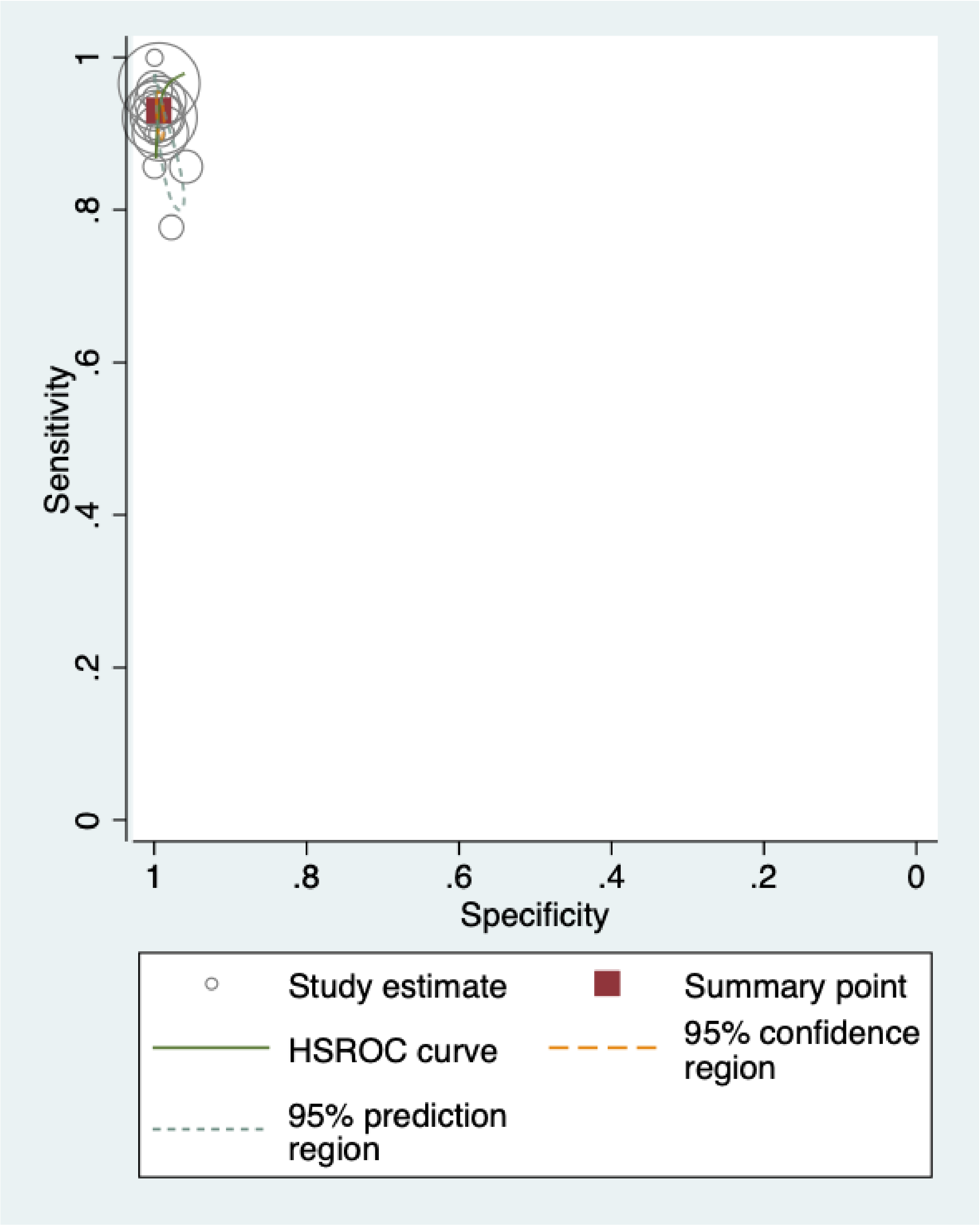

Supplementary Figure 2. Assessment for small study effects for multisite pooled testing for chlamydia.

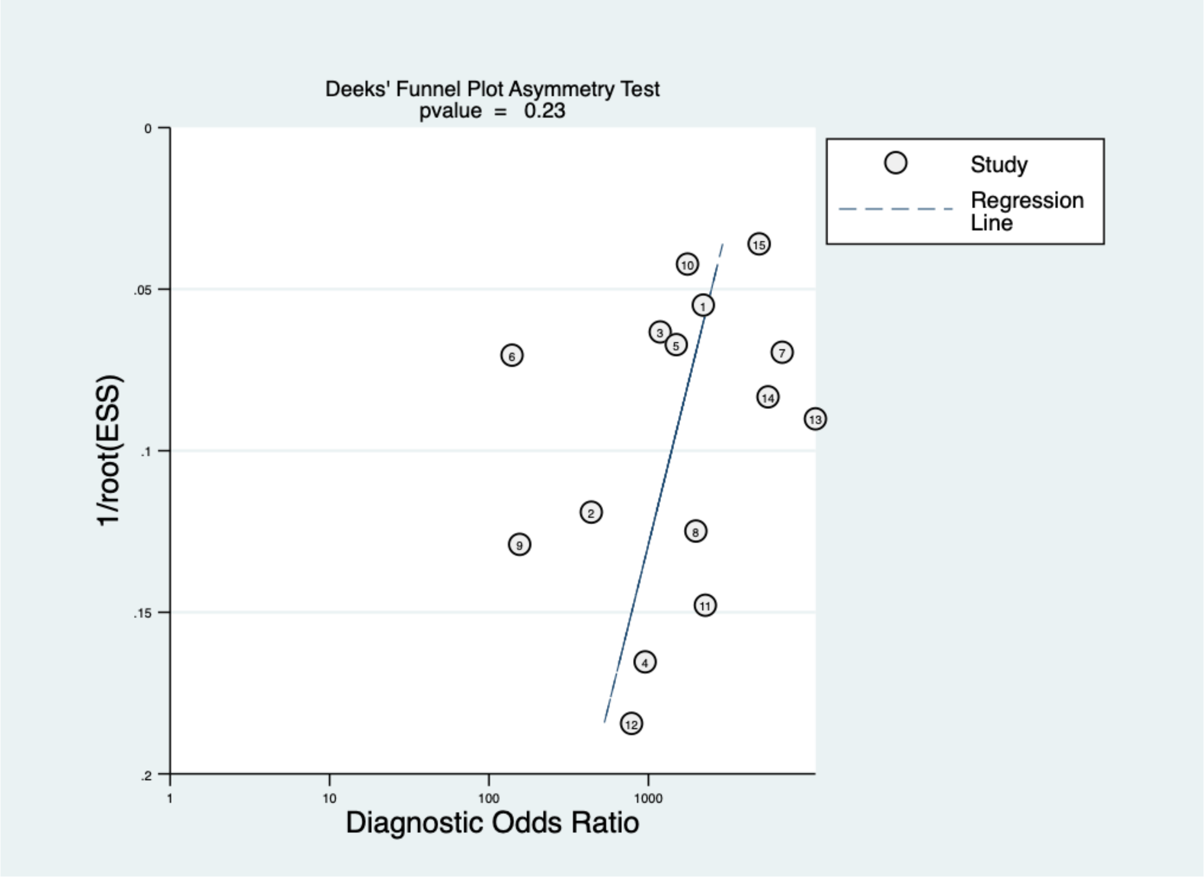

Supplementary Figure 3. Risk of Bias Summary as percentage

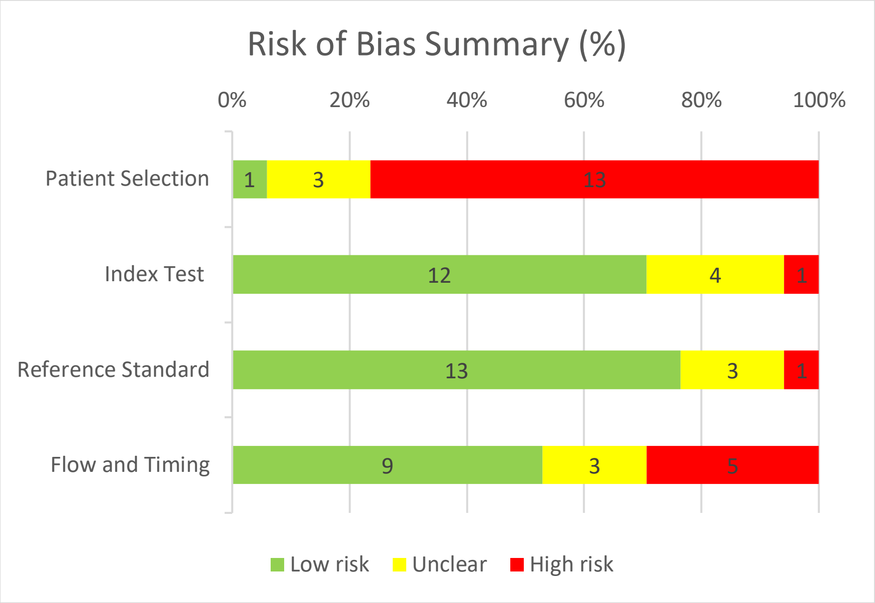

Supplementary Figure 4. Receiver operating characteristic (ROC) curve for multisite pooled testing for gonorrhoea.

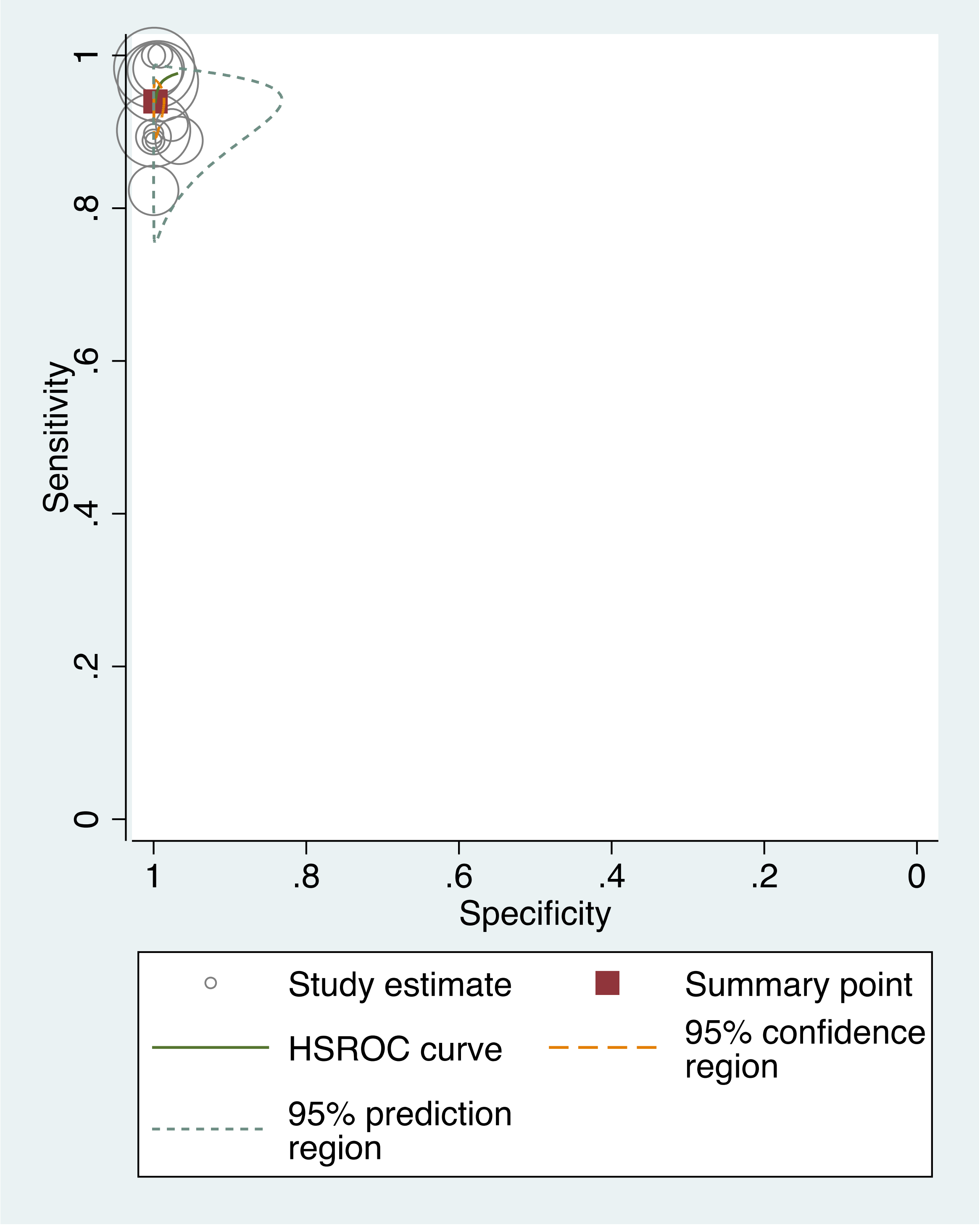

Supplementary Figure 5. Assessment for small study effects for multisite pooled testing for gonorrhoea.

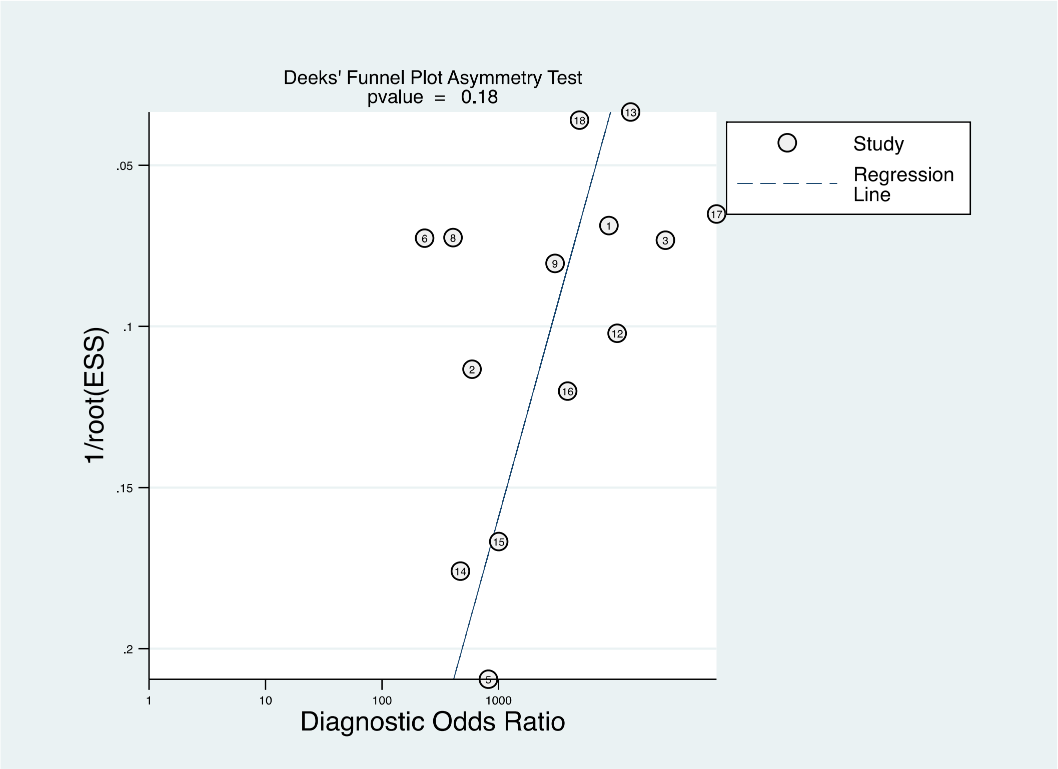

Supplementary Table 1. Meta-regression of the accuracy of multisite pooled testing for chlamydia according to study characteristics

Variable	Univariable			Multivariable		
	β (95% CI)	P-value	Adjusted R^2	β (95% CI)	P-value	Adjusted R^2
**SENSITIVITY**						
**Study population**			68.3%			19.7%
Non-MSM (ref)						
MSM	-0.8 (-1.6 to 0.0)	0.048		-1.0 (-2.5 to 0.5)	0.157	
**Study population size**			-11.9%			
Less than 100 (ref)						
100 or more	0.1 (-0.9 to 1.1)	0.881		0.4 (-1.4 to 2.2)	0.595	
**Country Income Level**			-7.4%			
High income (ref)						
Low- or Middle-income	0.5 (-0.5 to 1.5)	0.342		1.8 (-2.5 to 6.1)	0.325	
**Sample collection**			-22.0%			
Self-collected (ref)						
Clinician-collected	-0.2 (-1.4 to 1.1)	0.781		-0.6 (-2.2 to 1.0)	0.400	
**Publication year**			-20.2%			
Before 2020 (ref)						
2020 or after	0.2 (-0.6 to 1.0)	0.573		0.1 (-1.3 to 1.5)	0.848	
**SPECIFICITY**						
**Study population**			12.0%			-44.6%
Non-MSM (ref)						
MSM	-1.0 (-2.3 to 0.4)	0.145		-1.3 (-4.4 to 1.8)	0.342	
**Study population size**			-2.8%			
Less than 100 (ref)						
100 or more	0.6 (-0.9 to 2.0)	0.421		0.4 (-2.8 to 3.6)	0.756	
**Country Income Level**			-5.5%			
High income (ref)						
Low- or Middle-income	0.3 (-1.2 to 1.8)	0.648		0.0 (-5.7 to 5.7)	1.000	
**Sample collection**			-12.2%			
Self-collected (ref)						
Clinician-collected	0.4 (-1.5 to 2.4)	0.620		0.0 (-3.1 to 3.1)	0.999	
**Publication year**			-9.2%			
Before 2020 (ref)						
2020 or after	-0.1 (-1.4 to 1.1)	0.826		0.0 (-2.8 to 2.8)	0.999	

95% CI = 95% confidence intervals; MSM = men who have sex with men

Supplementary Table 2. The positive predictive value (PPV) and negative predictive value (NPV) for multisite pooled testing for chlamydia, over a range of background prevalence of chlamydia.

Prevalence	Sensitivity	Specificity	PPV	NPV	Number of cases	Missed cases	False Positive (Overtreated)
0.05	0.931	0.994	0.891	0.996	50	3	6
0.1	0.931	0.994	0.945	0.992	100	7	5
0.15	0.931	0.994	0.965	0.988	150	10	5
0.2	0.931	0.994	0.975	0.983	200	14	5
0.25	0.931	0.994	0.981	0.977	250	17	5
0.3	0.931	0.994	0.985	0.971	300	21	4
0.35	0.931	0.994	0.988	0.964	350	24	4
0.4	0.931	0.994	0.990	0.956	400	28	4
0.45	0.931	0.994	0.992	0.946	450	31	3
0.5	0.931	0.994	0.994	0.935	500	35	3
0.55	0.931	0.994	0.995	0.922	550	38	3
0.6	0.931	0.994	0.996	0.906	600	41	2
0.65	0.931	0.994	0.997	0.886	650	45	2
0.7	0.931	0.994	0.997	0.861	700	48	2
0.75	0.931	0.994	0.998	0.828	750	52	2
0.8	0.931	0.994	0.998	0.783	800	55	1
0.85	0.931	0.994	0.999	0.718	850	59	1
0.9	0.931	0.994	0.999	0.615	900	62	1
0.95	0.931	0.994	1.000	0.431	950	66	0
1	0.931	0.994	1.000	0.000	1000	69	0

Supplementary Table 3. Risk of Bias Summary

Study	Patient Selection	Index Test	Reference Standard	Flow and Timing
Ando[14]	+	+	+	+
Badman[7]	[a] −	+	+	[b] −
Bristow[11]	[a] −	[c] −	+	+
Chernesky[6]	[a] −	+	+	+
De Baetselier[2]	[a] −	+	+	[d] −
De Baetselier[1]	[e] !	[e] !	[e] !	+
Dean[8]	[e] !	+	+	[b] −
Durukan[3]	[a] −	+	+	+
Romyco[15]	[e] !	[e] !	[e] !	+
Shaw[16]	[a] −	[e] !	[e] !	[e] !
Singh[5]	[a] −	[e] !	+	[e] !
Speers[13]	[a] −	+	[f] −	+
Sultan[10]	[a] −	+	+	+
Thammajaruk[9]	[a] −	+	+	[g] −
Thielemans[12]	[a] −	+	+	[c] −
Verougstraete[17]	[a] −	+	+	+
Wilson[4]	[a] −	+	+	[e]

[a] Selection bias evident. Patients not enrolled in a randomised or consecutive fashion.
b If one or more anatomical specimens per participant provided a 'detected' result, all three individual specimens were then pooled and retested.
[c] Potential for decreased bacterial load in pooled specimens.
[d] Three sets excluded from analysis due to being incomplete.
[e] Not enough information.
[f] Pooled specimens tested by GeneXpert system, whilst un-pooled individual specimens tested by Cobas system. Re-testing of Cobas negative samples not undertaken.
[g] Only 50 of 199 patients had samples pooled. Patient characteristics of those selected not available.

Most studies scored "high" for risk of bias in the patient selection criterion as the nature of the study designs and context infers an automatically high risk of selection bias (i.e. patients were not randomised or recruited consecutively). However, the population selected should

have no significant effect on the sensitivity and specificity of NAAT. In studies by Badman et al.[7] and Dean et al.,[8] if one or more anatomic specimens per participant provided a 'detected' result on individual testing, all three samples from that patient were then pooled and retested. This excluded patients who may have attained false-negative results via individual testing and conferred a high risk of bias for study flow and timing. Studies by Bristow et al.[11] and Thielemans et al.[12] also had study designs that could decrease the bacterial load of pooled specimens and thus the diagnostic accuracy of the index test. For Bristow et al.,[11] pooled samples were created using remnant aliquots from un-pooled individual samples. Thielemans et al.[12] had all un-pooled individual samples taken before pooled samples. A study by de Baetselier et al.[2] excluded three pooled sets from their analysis due to their being labelled as "incomplete" with no explanation provided. The study by Speers et al.[13] used two different NAAT systems (GeneXpert and Cobas) to test pooled and un-pooled specimens, respectively, and thus scored a high risk of bias for their reference standard. In the study performed by Thammajaruk et al.,[9] 50 of the 199 enrolled patients had their samples pooled. The patient characteristics of those 50 selected for pooling was not available, and thus flow and timing were scored for a high risk of bias.

Supplementary Table 4. GRADE table for multisite pooled testing for chlamydia

Sensitivity	0.93 (95% CI: 0.91 to 0.95)
Specificity	0.99 (95% CI: 0.99 to 1.00)

Prevalences	0%	10%	20%

Outcome	№ of studies (№ of patients)	Study design	Factors that may decrease certainty of evidence					Effect per 1,000 patients tested			Test accuracy CoE
			Risk of bias	Indirectness	Inconsistency	Imprecision	Publication bias	pre-test probability of 0%	pre-test probability of 10%	pre-test probability of 20%	
**True positives** (patients with chlamydia)	14 studies 5891 patients	cross-sectional (cohort type accuracy study)	serious [a]	not serious	not serious	not serious	none	0 (0 to 0)	93 (91 to 95)	186 (181 to 190)	⊕⊕⊕◯ MODERATE
**False negatives** (patients incorrectly classified as not having chlamydia)								0 (0 to 0)	7 (5 to 9)	14 (10 to 19)	
**True negatives** (patients without chlamydia)	14 studies 5891 patients	cross-sectional (cohort type accuracy study)	serious [a]	not serious	not serious	not serious	none	994 (990 to 996)	895 (891 to 896)	795 (792 to 797)	⊕⊕⊕◯ MODERATE
**False positives** (patients incorrectly classified as having chlamydia)								6 (4 to 10)	5 (4 to 9)	5 (3 to 8)	

Explanations

a. Most studies had patient selection bias and some studies had potential for flow and timing bias.

Supplementary Table 5. Meta-regression of the accuracy of multisite pooled testing for gonorrhoea according to study characteristics

Variable	Univariable			Multivariable		
	β (95% CI)	P-value	Adjusted R^2	β (95% CI)	P-value	Adjusted R^2
**SENSITIVITY**						
**Study population**			-1.6%			-167.7%
Non-MSM (ref)						
MSM	-0.2 (-1.5 to 1.0)	0.667		0.6 (-2.1 to 3.3)	0.602	
**Study population size**			-19.4%			
Less than 100 (ref)						
100 or more	0.5 (-0.8 to 1.8)	0.426		1.4 (-1.9 to 4.6)	0.333	
**Country Income Level**			-14.6%			
High income (ref)						
Low- or Middle-income	-0.6 (-1.7 to 0.5)	0.271		0.0 (-4.6 to 4.6)	0.992	
**Sample collection**			-34.5%			
Self-collected (ref)						
Clinician-collected	-0.3 (-1.9 to 1.4)	0.746		-0.6 (-3.8 to 2.6)	0.653	
**Publication year**			-1.6%			
Before 2020 (ref)						
2020 or after	0.4 (-0.6 to 1.4)	0.407		0.7 (-1.9 to 3.3)	0.508	
**SPECIFICITY**						
**Study population**			7.2%			-61.7%
Non-MSM (ref)						
MSM	-1.0 (-2.8 to 0.7)	0.228		-1.0 (-4.0 to 2.0)	0.449	
**Study population size**			-1.5%			
Less than 100 (ref)						
100 or more	0.9 (-0.9 to 2.7)	0.302		0.7 (-2.8 to 4.2)	0.640	
**Country Income Level**			-5.8%			
High income (ref)						
Low- or Middle-income	-0.6 (-2.3 to 1.0)	0.415		0.1 (-4.8 to 5.1)	0.951	
**Sample collection**			-7.2%			
Self-collected (ref)						
Clinician-collected	0.6 (-1.3 to 2.6)	0.481		0.0 (-3.5 to 3.4)	0.983	
**Publication year**			-1.4%			
Before 2020 (ref)						
2020 or after	-0.5 (-2.0 to 1.0)	0.479		-0.2 (-0.6 to 11.6)	0.852	

95% CI = 95% confidence intervals; MSM = men who have sex with men

Supplementary Table 6. The positive predictive value (PPV) and negative predictive value (NPV) for multisite pooled testing for gonorrhoea, over a range of background prevalence of gonorrhoea.

Prevalence	Sensitivity	Specificity	PPV	NPV	Number of cases	Missed cases	False Positive (Overtreated)
0.05	0.941	0.996	0.925	0.997	50	3	4
0.1	0.941	0.996	0.963	0.993	100	6	4
0.15	0.941	0.996	0.976	0.990	150	9	3
0.2	0.941	0.996	0.983	0.985	200	12	3
0.25	0.941	0.996	0.987	0.981	250	15	3
0.3	0.941	0.996	0.990	0.975	300	18	3
0.35	0.941	0.996	0.992	0.969	350	21	3
0.4	0.941	0.996	0.994	0.962	400	24	2
0.45	0.941	0.996	0.995	0.954	450	27	2
0.5	0.941	0.996	0.996	0.944	500	30	2
0.55	0.941	0.996	0.997	0.932	550	32	2
0.6	0.941	0.996	0.997	0.918	600	35	2
0.65	0.941	0.996	0.998	0.901	650	38	1
0.7	0.941	0.996	0.998	0.879	700	41	1
0.75	0.941	0.996	0.999	0.849	750	44	1
0.8	0.941	0.996	0.999	0.808	800	47	1
0.85	0.941	0.996	0.999	0.749	850	50	1
0.9	0.941	0.996	1.000	0.652	900	53	0
0.95	0.941	0.996	1.000	0.470	950	56	0
1	0.941	0.996	1.000	0.000	1000	59	0

Supplementary Table 7. GRADE table for multisite pooled testing for gonorrhoea

Sensitivity	0.94 (95% CI: 0.91 to 0.96)
Specificity	1.00 (95% CI: 0.99 to 1.00)

Prevalences	0%	10%	20%

Outcome	№ of studies (№ of patients)	Study design	Factors that may decrease certainty of evidence					Effect per 1,000 patients tested			Test accuracy CoE
			Risk of bias	Indirectness	Inconsistency	Imprecision	Publication bias	pre-test probability of 0%	pre-test probability of 10%	pre-test probability of 20%	
**True positives** (patients with gonorrhoea)	13 studies 6565 patients	cross-sectional (cohort type accuracy study)	serious [a]	not serious	serious [b]	not serious	none	0 (0 to 0)	94 (91 to 96)	188 (182 to 193)	⊕⊕◯◯ LOW
**False negatives** (patients incorrectly classified as not having gonorrhoea)								0 (0 to 0)	6 (4 to 9)	12 (7 to 18)	
**True negatives** (patients without gonorrhoea)	13 studies 6565 patients	cross-sectional (cohort type accuracy study)	serious [a]	not serious	not serious	not serious	none	996 (991 to 998)	896 (892 to 898)	797 (793 to 798)	⊕⊕⊕◯ MODERATE
**False positives** (patients incorrectly classified as having gonorrhoea)								4 (2 to 9)	4 (2 to 8)	3 (2 to 7)	

Explanations

a. Most studies (n=13) had patient selection bias and 5 studies had potential for flow and timing bias.
b. Lower sensitivity noted in 4 studies for pharyngeal gonorrhoea

Supplementary Table 8. Study characteristics, methods of pooling, reported sensitivity and specificity of multisite pooled testing

Author	Country	Participants	Swabs used in pooling	Swab order	Individual swab taker	Pooled swab taker	Timing of pooling	Order of pooling	Volume of fluid in pooled sample	Total volume	NAAT used for pooled samples	NAAT used for individual samples	Sensitivity % (TP/ (TP+FN))	Specificity % (TN/ (TN+FP))
**Ando**[14]	Japan	513 MSM	Pharyngeal gargle, FVU, anorectal	Arbitrary done	Self collected	Self collected	Within 24 h of collection	Anorectal swab, gargle, FVU	1ml urine, 2ml gargle	3 ml	Hologic AC2	Hologic AC2	CT: 94.2% (98/104) NG: 98.3% (59/60)	CT: 99.3% (406/409) NG: 99.3% (450/453)
**Badman**[7]	Australia	388 MSM, 79 individuals pooled	Pharyngeal, anorectal, FVU	Only one pharyngeal and anorectal swab taken	Self collected	Self collected	All within 3 days	Urine added last	1 ml UTM from each pharyngeal and anorectal, 7 ml urine	9 ml	GeneXpert	GeneXpert	CT: 90.0% (45/50) NG: 89.7% (35/39)	N/A
**Bristow**[11]	US	644 MSM and transgender women	FVU, anorectal, pharyngeal	Only one pharyngeal and anorectal swab taken	Self collected	Self collected	N/A	Urine added last	0.34 ml from each sample	1.02 ml	Xpert CT/NG assay	Xpert CT/NG assay	CT: 90.1% (64/73) NG: 98.0% (50/51)	CT: 99.2% (519/523) NG: 99.8% (541/542)
**Chernesky**[6]	Canada	198 female	FVU, vaginal	Randomly chosen	Self collected	Self collected	Within 24 h once reaching the laboratory	Vaginal swab, urine	1 ml urine, undiluted original vaginal swab	1 ml	Hologic AC2	Hologic AC2	N/A	N/A
**De baetselier**[2]	Belgium	98 MSM	FVU, anorectal and pharyngeal	Randomly chosen	Self collected anorectal, clinician collected pharyngeal	Self collected anorectal, clinician collected pharyngeal	Upon arrival in laboratory	Urine first	170 µL each	510 µL	Abbott Real Time CT/GC assay	Abbott Real Time CT/GC assay	CT: 90.0% (9/10) NG: 100% (5/5)	CT: 99.1% (106/107) NG: 99.1% (111/112)
**De baetselier**[1]	West Africa	497 MSM	FVU, anorectal and pharyngeal	Randomly chosen	Physician collected anorectal and pharyngeal	Physician collected anorectal and pharyngeal	Upon arrival in laboratory	N/A	400 µL each	1200 µL	Cepheid GeneXpert	Abbott Real Time CT/GC assay	CT: 92.3% (60/65) NG: 88.9% (48/54)	CT: 99.2% (374/377) NG: 96.7% (377/390)
**Dean**[8]	Australia	253	FVU, anorectal and pharyngeal	N/A	N/A	N/A	N/A	N/A	1 ml each	3 ml	Xpert CT/NG assay	Xpert CT/NG assay	CT: 98.0% (48/49) NG: 93.2% (41/44)	N/A
**Durukan**[3]	Australia	162 MSM	FVU, anorectal and pharyngeal	Alternate with study id number	Research nurse collected anorectal and pharyngeal	Research nurse collected anorectal and pharyngeal	Within 24 h	Anorectal, pharyngeal, urine	2 ml urine	Unclear	Hologic AC2	Hologic AC2	CT: 85.7% (90/105) NG: 90.9 (70/77)	CT: 95.9% (93/97) NG: 97.6% (122/125)
**Romyco**[15]	Indonesia	251 MSM	FVU, anorectal	N/A	N/A	N/A	N/A	N/A	N/A	Unclear	GeneXpert	GeneXpert	CT: 95.9% (70/73)	CT: 100% (171/171)

			and pharyngeal										NG: 89.4% (42/47)	NG: 100% (197/197)
**Singh**[5]	UK	99 Symptomatic + asymptomatic female <25 years	Genital, anorectal, pharyngeal	N/A	Either clinician or self collected	Either clinician or self collected	N/A	N/A	N/A	Unclear	AC2	AC2	CT: 94.7% (18/19)	CT: 100% (80/80)
**Speers**[13]	Australia	107 MSM	FVU, anorectal and pharyngeal	Dual headed swabs	Clinician collected pharyngeal, self collected anorectal	Clinician collected pharyngeal, self collected anorectal	Upon arrival in laboratory	N/A	7 ml urine	Unclear	Cepheid GeneXpert	Roche Cobas 4800	CT: 77.8% (14/18) NG: 100% (34/34)	CT: 97.8% (89/91) NG: 100% (75/75)
**Sultan**[10]	UK	1064 MSM	FVU, anorectal and pharyngeal	Randomise d	Clinician collected	self collected	Once samples taken	Method A: urine first, Method B: urine last	Method B: 2 ml urine added in undiluted original swabs	2ml	Hologic AC2	Hologic AC2	CT: 92.2% (153/166) NG: 89.2% (257/288)	CT: 99.3% (897/903) NG: 99.5% (777/781)
**Thammaja ruk**[9]	Thailand	199 MSM and transgender women, pooled samples from 50	FVU, anorectal and pharyngeal	N/A	Lay health provider collected pharyngeal and anorectal	Lay health provider collected pharyngeal and anorectal	N/A	N/A	N/A	Unclear	Cepheid GeneXpert	Cepheid GeneXpert	CT: 100% (16/16) NG: 88.9% (8/9)	CT: 100% (34/34) NG: 100% (41/41)
**Thieleman s**[12]	Belgium	100 MSM	FVU, anorectal and pharyngeal	Unpooled samples were taken first	Clinician collected pharyngeal, self collected anorectal	Clinician collected pharyngeal, self collected anorectal	once samples taken	urine last	3 ml urine	Unclear	Abbott Real Time	Abbott Real Time	CT: 85.7% (6/7) NG: 88.9% (8/9)	CT: 100% (90/90) NG: 100% (88/88)
**Verougstra ete**[17]	Belgium	489 female sex workers	Pharyngeal, anorectal, vaginal	Only one taken each	Physician collected pharyngeal, either self or physician collected vaginal and anorectal	Physician collected pharyngeal, either self or physician collected vaginal and anorectal	N/A	N/A	400 µL each	1200 µL	Abbott Real Time CT/GC assay	Abbott Real Time CT/GC assay	CT: 93.7% (30/32) NG: 82.4% (14/17)	CT: 100% (457/457) NG: 100% (472/472)
**Wilson**[4]	UK	1284 women, 509 MSM	Pharyngeal, anorectal, FVU in MSM, VVS in women	Randomise d	Both self and clinician collected	Self collected	Once samples taken	Pharyngeal, anorectal then VVS in women or FVU in MSM	2 ml urine in MSM, no urine sample in women	Unclear	Hologic AC2	Hologic AC2	Women: CT: 96.6% (229/237) NG: 98.4% (61/62) MSM:	Women: CT: 99.4% (1038/1044) NG: 99.9% (1218/1219) MSM:

CT: 92.3% (36/39)<br>NG: 98.1% (51/52)	CT: 99.8% (469/470)<br>NG: 99.6% (455/457)

FN = false negative, FP = false positive, TN = true negative, TP= true positive
FVU = first void urine, MSM = men who have sex with men, N/A = not applicable, VVS = vulvovaginal swab

Sixteen studies provided details about their methodology of multisite pooled testing (Supplementary Table 8). Nine studies stated the pooling order but no standard order was shared across them. Similarly, there was a lack of consistency in the amount of urine added into the pooled sample, which can act as a dilutant. The amount varied from 170 μL to 7 mL, and only two papers[3,7] commented on the effect of urine on diagnostic accuracy. Badman et al. suggested the possibility of overdiluting the bacterial DNA load in the pooled sample given many participants only had an extragenital infection and their additional pooling experiment showed a slight improvement in diagnostic accuracy when reducing the urine input from 7 mL to 1 mL.[7] Durukan et al. proposed that low load infections can be undetectable with urine dilution, but the effect of various urine volumes was not tested in their study.[3] The majority of studies (n=12) collected first void urine (FVU), pharyngeal and anorectal specimen for both pooling and individual testing, whilst three studies utilised genital swabs instead of FVU and Chernesky's study only pooled vaginal and urine samples instead of triple site pooling[6].
