# Supplementary material for: Diagnostic accuracy of pooling urine, anorectal, and oropharyngeal specimens for the detection of Chlamydia trachomatis and Neisseria gonorrhoeae: a systematic review and meta-analysis
Source: BMC Med. 2021 Nov 25;19:285. doi: 10.1186/s12916-021-02160-9 (PMC8614052; doi:10.1186/s12916-021-02160-9)
Supplement: Supplementary file 1 — Additional file 1: Appendix 1. Search strategy. Appendix 2. Further details of multisite pooled sampling for chlamydia and gonorrhoea. Supplementary Figure 1. Receiver operating characteristic (ROC) curve for multisite pooled testing for chlamydia. Supplementary Figure 2. Assessment for small study effects for multisite pooled testing for chlamydia. Supplementary Figure 3. Risk of bias summary as percentage. Supplementary Figure 4. Receiver operating characteristic (ROC) curve for multisite pooled testing for gonorrhoea. Supplementary Figure 5. Assessment for small study effects for multisite pooled testing for gonorrhoea. Supplementary Table 1. Meta-regression of the accuracy of multisite pooled testing for chlamydia according to study characteristics. Supplementary Table 2. The positive predictive value (PPV) and negative predictive value (NPV) for multisite pooled testing for chlamydia, over a range of background prevalence of chlamydia. Supplementary Table 3. Risk of Bias summary. Supplementary Table 4. GRADE table for multisite pooled testing for chlamydia. Supplementary Table 5. Meta-regression of the accuracy of multisite pooled testing for gonorrhoea according to study characteristics. Supplementary Table 6. The positive predictive value (PPV) and negative predictive value (NPV) for multisite pooled testing for gonorrhoea, over a range of background prevalence of gonorrhoea. Supplementary Table 7. GRADE table for multisite pooled testing for gonorrhoea. Supplementary Table 8. Study characteristics, methods of pooling, reported sensitivity and specificity of multisite pooled testing. References. [file 12916_2021_2160_MOESM1_ESM.zip › Additional file 1 References.docx]

**Additional File 1: References**

1. De Baetselier I, Vuylsteke B, Yaya I, et al. To pool or not to pool samples for sexually transmitted infections detection in men who have sex with men? An evaluation of a new pooling method using the genexpert instrument in West Africa. *Sexually Transmitted Diseases* 2020; **47**(8): 556-61.

2. De Baetselier I, Osbak KK, Smet H, Kenyon CR, Crucitti T. Take three, test one: a cross-sectional study to evaluate the molecular detection of Chlamydia trachomatis and Neisseria gonorrhoeae in pooled pharyngeal, anorectal and urine samples versus single-site testing among men who have sex with men in Belgium. *Acta Clinica Belgica* 2020; **75**(2): 91-5.

3. Durukan D, Read TRH, Bradshaw CS, et al. Pooling Pharyngeal, Anorectal, and Urogenital Samples for Screening Asymptomatic Men Who Have Sex with Men for Chlamydia trachomatis and Neisseria gonorrhoeae. *Journal of Clinical Microbiology* 2020; **58**(5): 23.

4. Wilson JD, Wallace HE, Loftus-Keeling M, et al. Swab-yourself trial with economic monitoring and testing for infections collectively (SYSTEMATIC): Part 2. A diagnostic accuracy, and cost-effectiveness, study comparing rectal, pharyngeal and urogenital samples analysed individually, versus as a pooled specimen, for the diagnosis of gonorrhoea and chlamydia. *Clinical Infectious Diseases* 2020; **12**: 12.

5. Singh S, Hopkins M, Sarner L. High prevalence of extragenital chlamydia trachomatis (CT) in heterosexual women: Validation of pooled samples. *Sexually Transmitted Infections* 2019; **95 (Supplement 1)**: A217-A8.

6. Chernesky M, Jang D, Martin I, et al. Mycoplasma genitalium, Chlamydia trachomatis, and Neisseria gonorrhoeae Detected With Aptima Assays Performed on Self-Obtained Vaginal Swabs and Urine Collected at Home and in a Clinic. *Sexually Transmitted Diseases* 2019; **46**(9): e87-e9.

7. Badman SG, Bell SFE, Dean JA, et al. Reduced sensitivity from pooled urine, pharyngeal and rectal specimens when using a molecular assay for the detection of chlamydia and gonorrhoea near the point of care. *Sexual Health* 2020; **17**(1): 15-21.

8. Dean JA, Bell SFE, Coffey L, et al. Improved sensitivity from pooled urine, pharyngeal and rectal specimens when using a molecular assay for the detection of chlamydia and gonorrhoea near point of care. *Sexually Transmitted Infections* 2020; **13**: 13.

9. Thammajaruk N, Promthong S, Posita P, et al. Pooled pharyngeal, rectal and urine samples for the Point-of-Care detection of Chlamydia trachomatis and Neisseria gonorrhoeae by lay-providers in Key Population-Led Health Services in Thailand. *Journal of the International Aids Society* 2020; **23**: 115-6.

10. Sultan B, White JA, Fish R, et al. The "3 in 1" Study: Pooling Self-Taken Pharyngeal, Urethral, and Rectal Samples into a Single Sample for Analysis for Detection of Neisseria gonorrhoeae and Chlamydia trachomatis in Men Who Have Sex with Men. *Journal of Clinical Microbiology* 2016; **54**(3): 650-6.

11. Bristow CC, Mehta SR, Hoenigl M, Little SJ. The performance of pooled 3-anatomic-site chlamydia and gonorrhea testing. *Topics in Antiviral Medicine* 2020; **28 (1)**: 398-9.

12. Thielemans E, Wyndham-Thomas C, Henrard S, et al. Screening for Chlamydia trachomatis and Neisseria gonorrhoeae Infections in Men Who Have Sex With Men: Diagnostic Accuracy of Nucleic Acid Amplification Test on Pooled Urine, Anorectal, and Pharyngeal Specimens. *Sexually Transmitted Diseases* 2018; **45**(3): 195-8.

13. Speers DJ, Chua IJ, Manuel J, Marshall L. Detection of Neisseria gonorrhoeae and Chlamydia trachomatis from pooled rectal, pharyngeal and urine specimens in men who have sex with men. *Sexually Transmitted Infections* 2018; **94**(4): 293-7.

14. Ando N, Mizushima D, Watanabe K, et al. Modified self-obtained pooled sampling to screen for Chlamydia trachomatis and Neisseria gonorrhoeae infections in men who have sex with men. *Sexually Transmitted Infections* 2020; **20**: 20.

15. Romyco I, Umboh L, Erari S, Wignall S. Performance of 3-in-1 pooled samples from anal, rectal, and throat of genexpert CT/NG in Bali, Indonesia. *Sexually Transmitted Infections* 2019; **95 (Supplement 1)**: A104.

16. Shaw J, Saunders JM, Hughes G. Attitudes to, and experience of, pooled sampling for sexually transmitted infection testing: a web-based survey of English sexual health services. *International Journal of STD & AIDS* 2018; **29**(6): 547-51.

17. Verougstraete N, Verbeke V, De Canniere AS, Simons C, Padalko E, Coorevits L. To pool or not to pool? Screening of Chlamydia trachomatis and Neisseria gonorrhoeae in female sex workers: pooled versus single-site testing. *Sexually Transmitted Infections* 2020; **96**(6): 417-21.
